# Supplementary material for: Tracing the origin and evolution of supergene mimicry in butterflies
Source: Nat Commun. 2017 Nov 7;8:1269. doi: 10.1038/s41467-017-01370-1 (PMC5677128; doi:10.1038/s41467-017-01370-1)
Supplement: Supplementary file 1 — Supplementary Information [file 41467_2017_1370_MOESM1_ESM.pdf]

Supplementary Table 1. Sample information and sequencing statistics

Sample	Taxon	Morph	Sex	Collecting location	Raw Reads (Gb)	Filtered Reads (Gb)	Mapped rate (%)	Genotype calls (Qual≥30)	Mean Depth (Qual≥30)
PR345	*Papilio Polytes*		male	lab reared	4.76	4.66	90.43	36236144	15.85
PR346	*Papilio Polytes*		male	lab reared	2.40	2.35	91.07	35233509	8.01
PR347	*Papilio Polytes*		male	lab reared	3.81	3.73	90.81	36107778	12.93
PR348	*Papilio Polytes*		male	lab reared	3.60	3.52	90.21	35719296	12.46
PR349	*Papilio Polytes*		male	lab reared	4.55	4.45	90.95	36441209	15.01
PR350	*Papilio Polytes*	*cyrus*	female	lab reared	2.56	2.51	90.54	35464909	8.94
PR351	*Papilio Polytes*	*cyrus*	female	lab reared	2.99	2.93	90.56	35858619	10.26
PR352	*Papilio Polytes*	*cyrus*	female	lab reared	2.71	2.66	90.38	35405401	9.41
PR353	*Papilio Polytes*	*cyrus*	female	lab reared	2.97	2.90	90.71	35591372	10.07
PR354	*Papilio Polytes*	*cyrus*	female	lab reared	3.08	3.01	90.27	35602801	10.59
PR355	*Papilio Polytes*	*cyrus*	female	lab reared	1.80	1.74	90.41	33665981	6.44
PR356	*Papilio Polytes*	*cyrus*	female	lab reared	3.33	3.23	81.07	35339553	10.37
PR357	*Papilio Polytes*	*cyrus*	female	lab reared	2.90	2.81	90.67	35516315	10.08
PR358	*Papilio Polytes*	*cyrus*	female	lab reared	2.44	2.38	80.62	34431188	7.74
PR359	*Papilio Polytes*	*cyrus*	female	lab reared	2.05	1.98	90.59	34486129	7.16
PR360	*Papilio Polytes*		male	lab reared	2.02	1.83	54.79	32745479	4.16
PR361	*Papilio Polytes*		male	lab reared	3.30	3.20	90.51	35457278	11.54
PR362	*Papilio Polytes*		male	lab reared	2.00	1.94	90.99	34230653	7.05
PR363	*Papilio Polytes*		male	lab reared	2.33	2.25	91.2	35041142	7.99
PR364	*Papilio Polytes*		male	lab reared	1.61	1.56	83.43	33986445	5.28
PR365	*Papilio Polytes*	*polytes*	female	lab reared	1.68	1.62	91.18	34199491	5.85

PR366	*Papilio Polytes*	*polytes*	female	lab reared	2.09	2.02	86.17	34361994	7.19
PR367	*Papilio Polytes*	*polytes*	female	lab reared	2.88	2.79	90.76	35732553	10.08
PR368	*Papilio Polytes*	*polytes*	female	lab reared	1.98	1.92	91.15	34772512	6.83
PR369	*Papilio Polytes*	*polytes*	female	lab reared	1.99	1.92	90.88	34852610	6.99
PR370	*Papilio Polytes*	*polytes*	female	lab reared	2.16	2.09	90.79	35056121	7.50
PR371	*Papilio Polytes*	*polytes*	female	lab reared	2.01	1.94	91.05	34995597	6.94
PR372	*Papilio Polytes*	*polytes*	female	lab reared	2.90	2.81	90.58	35507931	10.03
PR373	*Papilio Polytes*	*polytes*	female	lab reared	2.18	2.11	90.14	34345076	7.70
PR374	*Papilio Polytes*	*polytes*	female	lab reared	2.83	2.73	90.46	35249732	9.72
pol_t6	*Papilio Polytes*	*theseus*	female	lab reared	8.44	7.85	89.59	37440311	23.77
pol_t7	*Papilio Polytes*	*theseus*	female	lab reared	2.74	2.66	89.87	36097594	8.84
timor1	*Papilio Polytes*	*polytes*	female	Timor, Indonesia	3.47	3.41	35.07	32279952	4.92
timor2	*Papilio Polytes*	*cyrus*	female	Timor, Indonesia	2.78	2.69	83.94	37707690	8.83
timor3	*Papilio Polytes*	*cyrus*	female	Timor, Indonesia	3.56	3.49	25.54	28239839	3.82
landak1	*Papilio Polytes*	*theseus*	female	Landak, Indonesia	3.76	3.56	91.82	38555717	12.32
lombok1	*Papilio Polytes*	modified *theseus*	female	Lombok, Indonesia	5.51	5.36	92.77	38685234	17.32
lombok2	*Papilio Polytes*	modified *theseus*	female	Lombok, Indonesia	5.18	4.98	83.32	38774944	14.87
lombok3	*Papilio Polytes*	*theseus*	female	Lombok, Indonesia	3.99	3.81	92.81	38644420	13.62
lombok4	*Papilio Polytes*	*theseus*	female	Lombok, Indonesia	3.92	3.76	86.56	38170099	11.81
sumatra1	*Papilio Polytes*		male	Sumatra, Indonesia	5.75	5.63	23.22	32831474	4.98
sumatra2	*Papilio Polytes*		male	Sumatra, Indonesia	2.21	2.09	95.1	37927337	8.24
jambi1	*Papilio Polytes*	*theseus*	female	Jambi, Indonesia	3.06	2.92	78.73	37794856	9.31
bali1	*Papilio Polytes*	*theseus*	female	Bali, Indonesia	3.35	3.25	90.86	36880721	10.37

romulus1	*Papilio Polytes*	*romulus*	female	Bangarupalyam Mandal, India	8.73	8.02	91.14	39316440	29.22
phestus1	*Papilio phestus*		male	Buka, Indonesia	3.94	3.59	32.68	24285031	3.14
phestus2	*Papilio phestus*		male	Buka, Indonesia	3.61	2.74	60.44	31393030	5.84
phestus3	*Papilio phestus*		male	Buka, Indonesia	12.24	10.38	32.51	34577294	8.82
phestus4	*Papilio phestus*		male	Buka, Indonesia	2.15	1.44	81.61	30199792	4.37
arfak1	*Papilio ambrax*		female	Arfak, Indonesia	3.05	2.65	77.9	25730404	6.05
arfak2	*Papilio ambrax*		female	Arfak, Indonesia	7.41	6.91	60.74	29512953	10.07
arfak3	*Papilio ambrax*		female	Arfak, Indonesia	5.66	5.24	84.55	35013294	13.31
arfak4	*Papilio ambrax*		female	Arfak, Indonesia	4.84	4.48	78.5	32887779	10.66
timika1	*Papilio ambrax*		female	Timika, Indonesia	1.98	1.49	87.69	28929884	4.64
timika2	*Papilio ambrax*		female	Timika, Indonesia	3.43	2.48	78.15	31387988	5.89
timika3	*Papilio ambrax*		female	Timika, Indonesia	3.75	2.85	86.58	33186526	7.42
timika4	*Papilio ambrax*		female	Timika, Indonesia	3.39	2.21	87.26	30287624	5.84
protenor1	*Papilio protenor*		female	Guilin, China	5.36	4.40	74.93	31709831	12.65
protenor2	*Papilio protenor*		female	Guilin, China	6.41	5.53	80.93	32590680	16.72
protenor3	*Papilio protenor*		male	Guilin, China	6.52	5.51	56.6	31140423	11.85
protenor4	*Papilio protenor*		male	Guilin, China	11.83	10.44	76.57	33512723	28.49

Supplementary Table 2. Individual genotypes of different species

	MM	Mm	mm
P. polytes polytes	1	10	2
P. polytes alphenor	15	2	15
P. ambrax and *P. phestus*	12	0	0

MM denotes homozygous mimetic

mm denotes homozygous non-mimetic

Mm denotes heterozygous

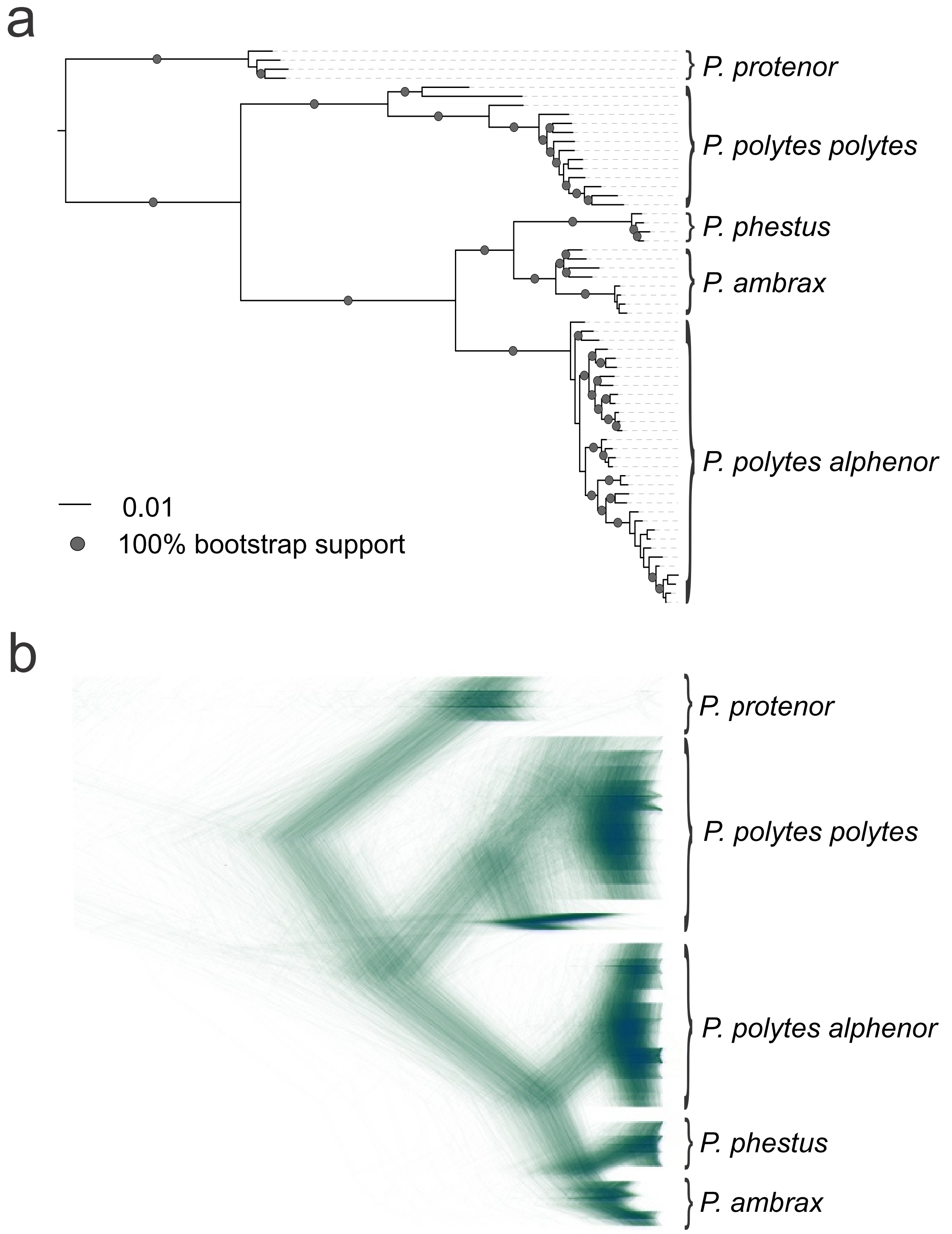

Supplementary Figure 1. Genome-wide phylogeny of *Papilio* butterflies.
(a) A maximum-likelihood phylogenetic tree based on 40.2 million genome-wide SNPs. (b) DensiTree of the polytes group produced by overlaying ML trees from 2,134 non-overlapping 100 kbp windows across the genome. 86% of the windows produce an identical tree as the full genome-wide tree, and all trees, except 1, place *P. polytes alphenor* as the sister lineage to *ambrax/phestus*.

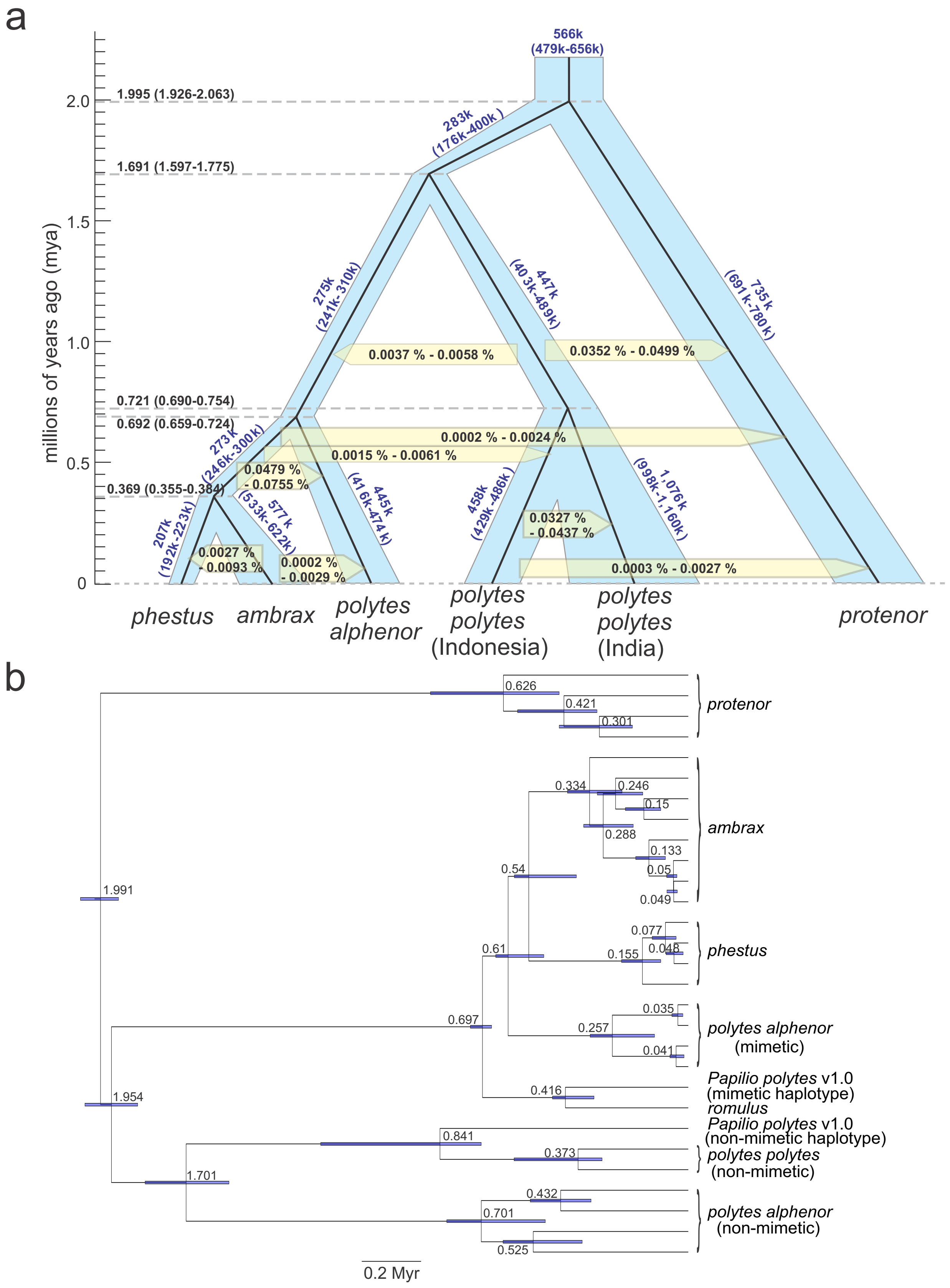

Supplementary Figure 2. Timing diversification events in the *Papilio polytes* group.
(a) Divergence times, effective population sizes, and migration rates were inferred among *polytes* group taxa using G-PhoCS. Branch widths are proportional to population size and dashed lines indicate divergence times. Parameter estimates are shown along with 95% Bayesian credible intervals. (b) Maximum-likelihood tree of *doublesex* haplotypes, calibrated using the G-PhoCS estimate of 1.995 ± 0.068 My ago for the common ancestor between *P. protenor* and the *polytes* group. Numbers are in million years and blue bars represent 95% confidence intervals. Comparison of (a) and (b) suggests that divergence between mimetic and non-mimetic *dsx* haplotypes (1.954 My ago) occurred before the radiation of the *polytes* group (1.691 My ago). Data are presented as mean ± SD.

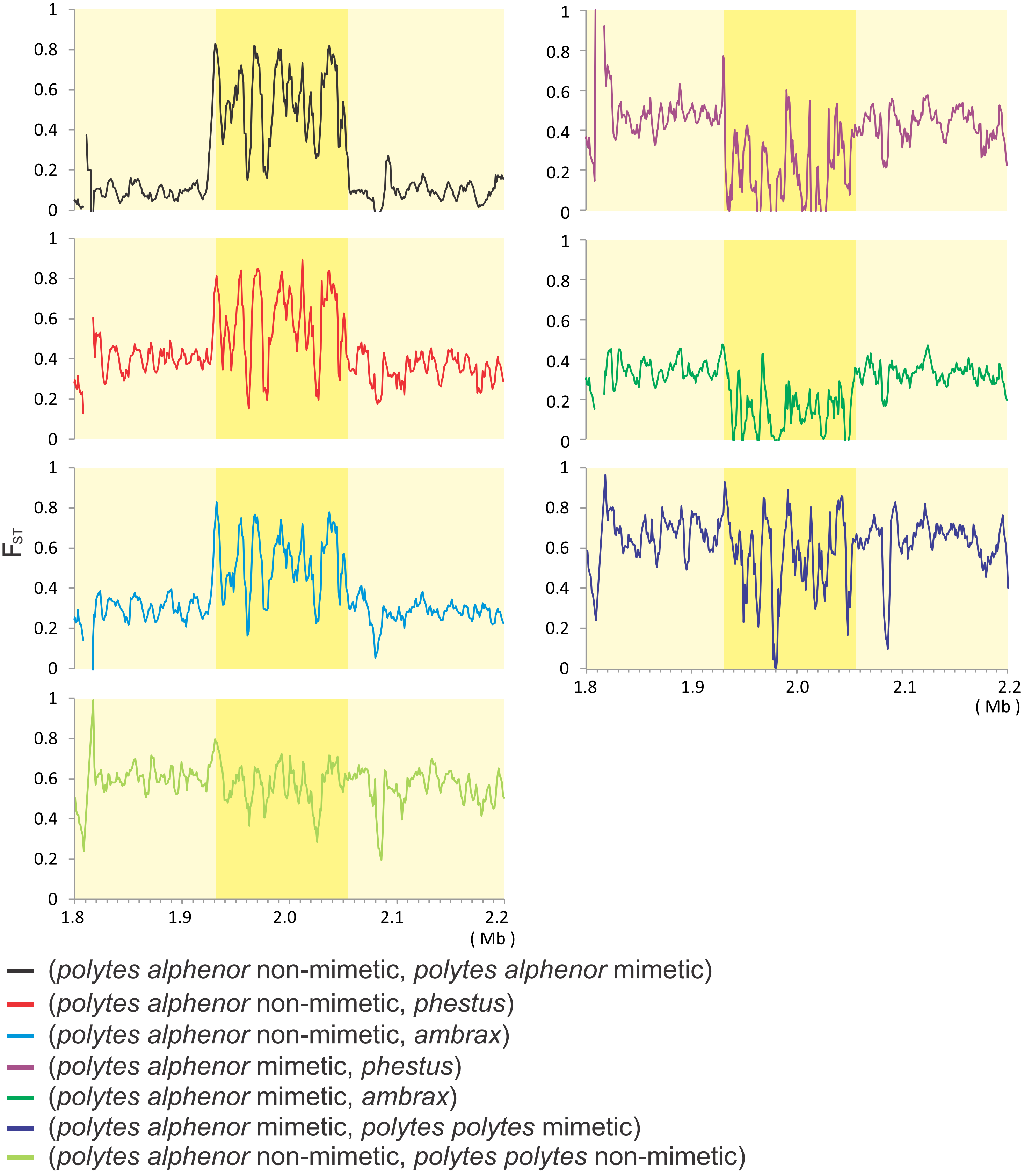

Supplementary Figure 3. Genetic differentiation (F_{ST}) along the *dsx* gene region.
F_{ST} scans were performed using a 5 kbp window size to infer the breakpoints of the *dsx* inversion in various species comparisons. The entire 130 kbp *dsx* inversion region is colored in darker yellow. Clear deviations, coinciding with inversion breakpoints, are found in the same locations in comparisons between *P. polytes alphenor* non-mimetic and *P. polytes alphenor* mimetic, between *P. polytes alphenor* non-mimetic and *P. ambrax*, between *P. polytes alphenor* non-mimetic and *P. phestus*, between *P. polytes alphenor* mimetic and *P. polytes polytes* mimetic and between *P. polytes alphenor* non-mimetic and *P. polytes polytes* non-mimetic.

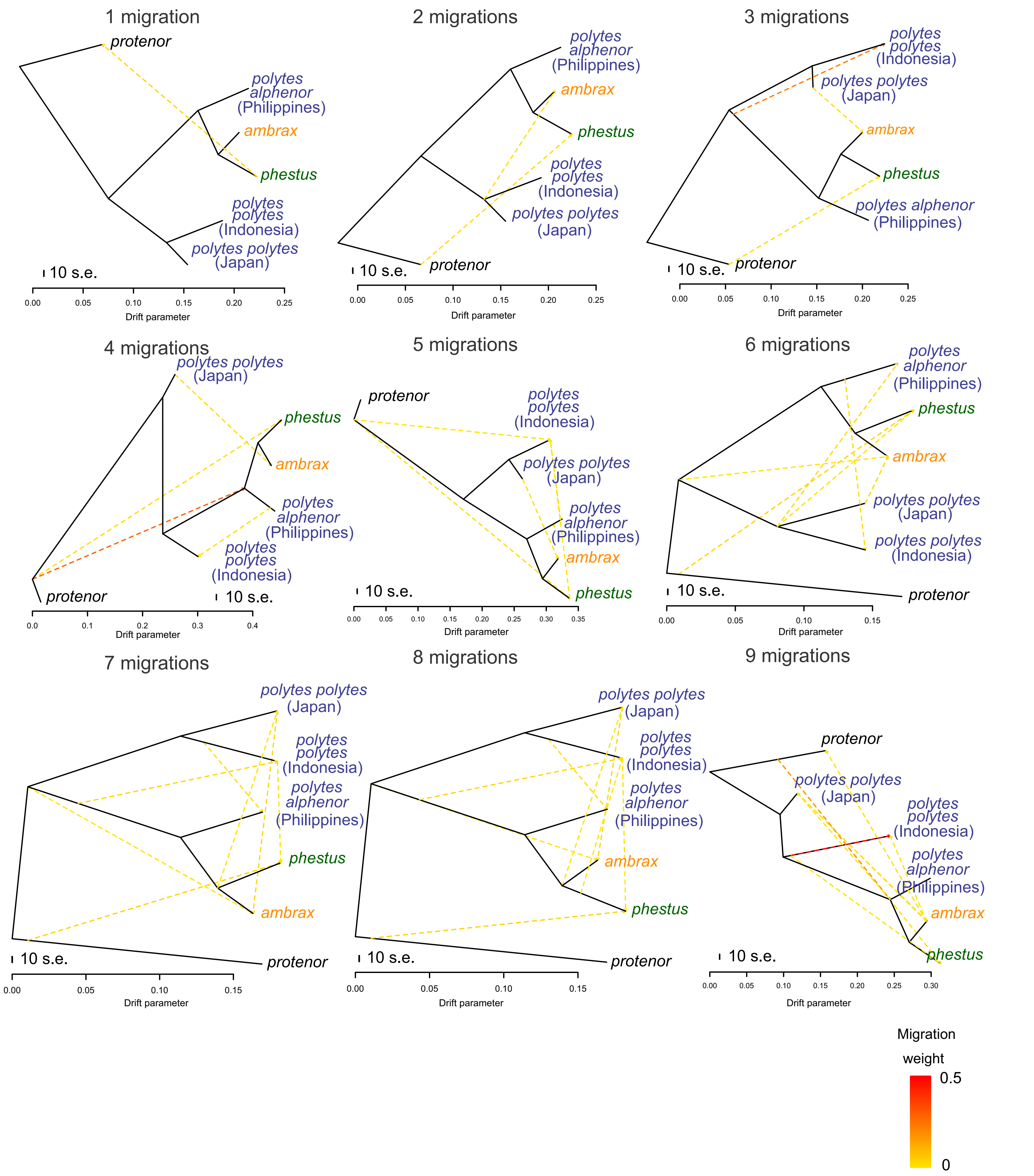

Supplementary Figure 4. Inferences of relationships and migration among *Papilio* species using Treemix. In each case, a maximum-likelihood tree was inferred among five species/populations in the *polytes* group, as well as the outgroup species *P. protenor*, based on genome-wide SNP allele frequency data. Migration was explored by assuming 1 to 9 migration edges.

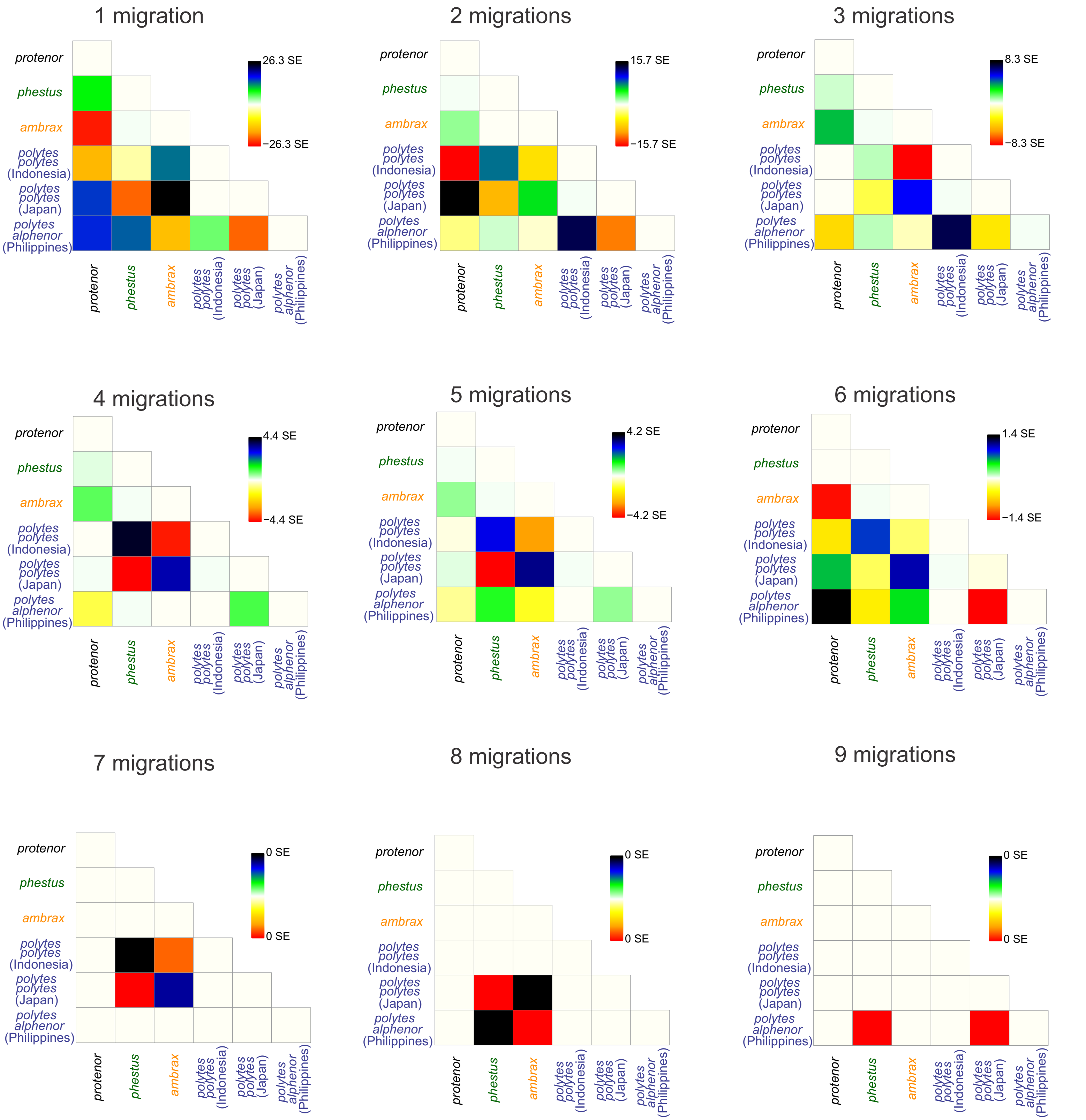

Supplementary Figure 5. Residue fit from the trees generated using Treemix.
Each residue fit matrix is generated by dividing the residual covariance of pairwise populations by the average standard error using Treemix. Residues above zero indicate pairs of populations that are closer to each other and could be candidates for migration events. Migration was explored by assuming 1 to 9 migration edges.

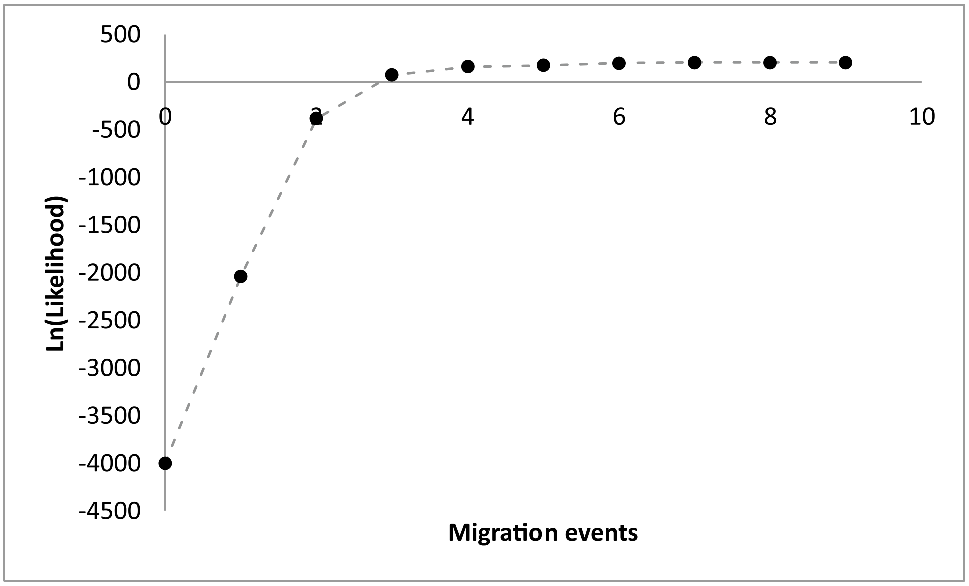

Supplementary Figure 6. Log-likelihood values for Treemix trees.
The Ln-likelihood values are calculated for Treemix trees by assuming 1 to 9 migration edges.

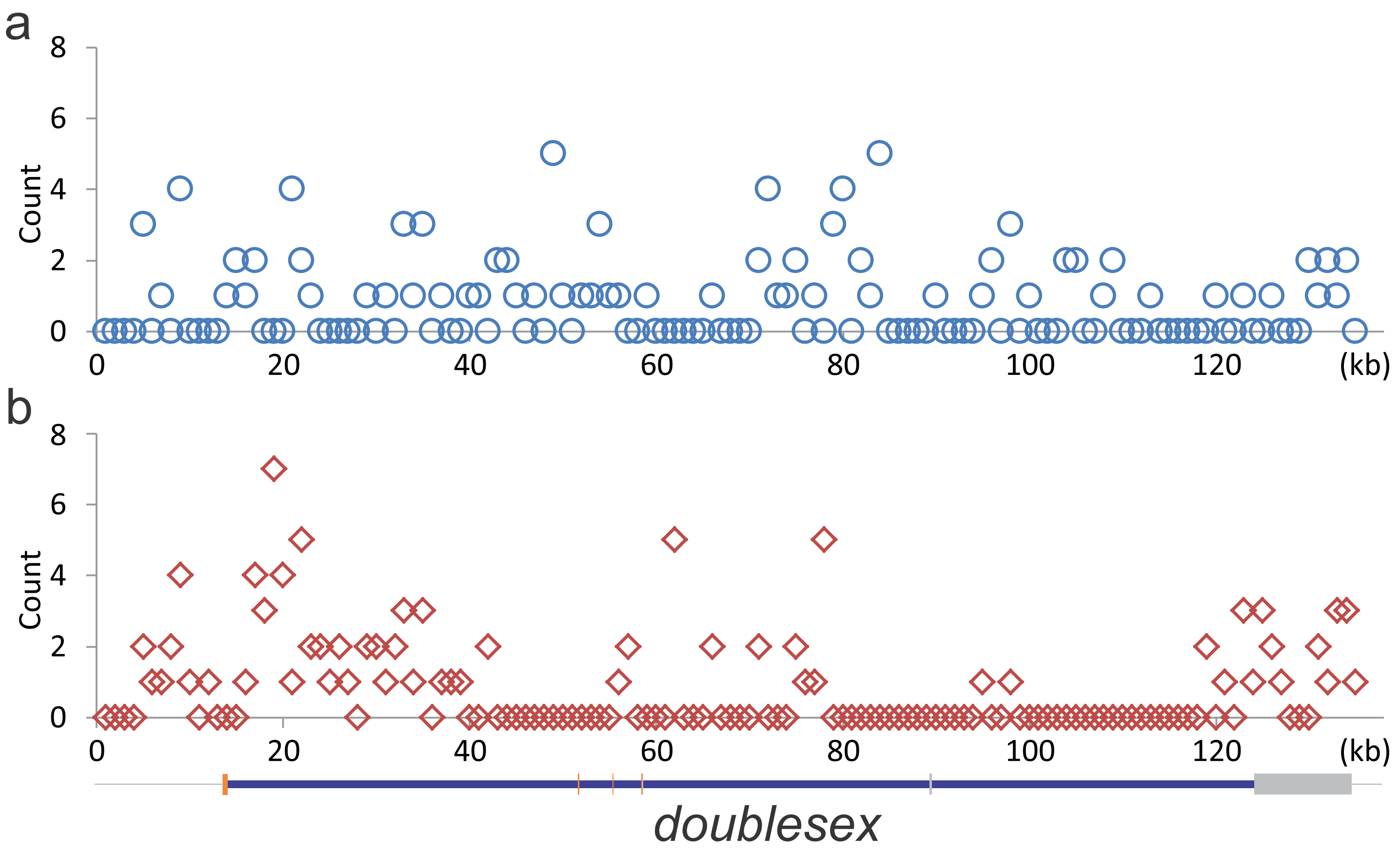

Supplementary Figure 7. ***Theseus*** **morph SNP counts across** ***doublesex*****.**
The number of *theseus* specific alleles are counted for Philippine group (a) or Indonesia group (b), independently, for each fixed 1 kbp window along the *dsx* gene and neighboring regions. The blue line represents the *dsx* gene. The orange and grey bars represent the coding sequence (CDS) and UTR regions, respectively.

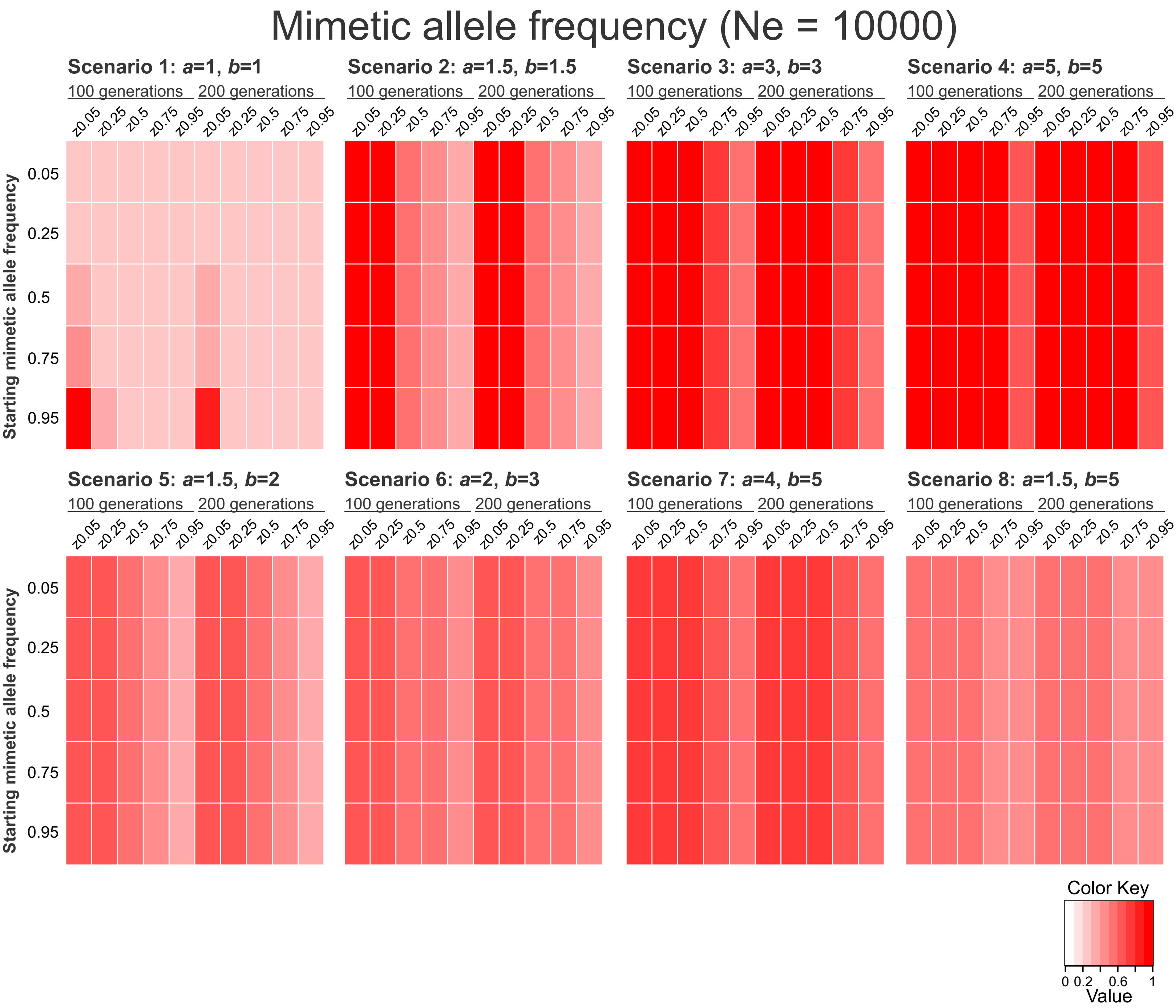

Supplementary Figure 8. Testing parameter sets for a modified model of negative-frequency-dependent-selection (NFDS).
A number of a and b values were selected to model different scenarios, including pure NFDS ($a = b = 1$), modified NFDS with equal benefits to homozygous mimetic and heterozygous genotypes ($a = b =1.5$, 3 and 5) and modified NFDS with heterozygote advantage ($a = 1.5$ $b = 2$, $a = 2$ $b = 3$, $a = 4$ $b = 5$ and $a = 1.5$ $b = 5$). The starting mimetic allele frequency ranged from 0.05 to 0.95. Different z values indicate different strengths of NFDS, from neutral ($z = 0$) to very strong ($z = 0.95$). Each grid represents the mean mimetic allele frequency from 10 separate runs of each model after 100 or 200 generations, with a constant population size Ne = 10,000.

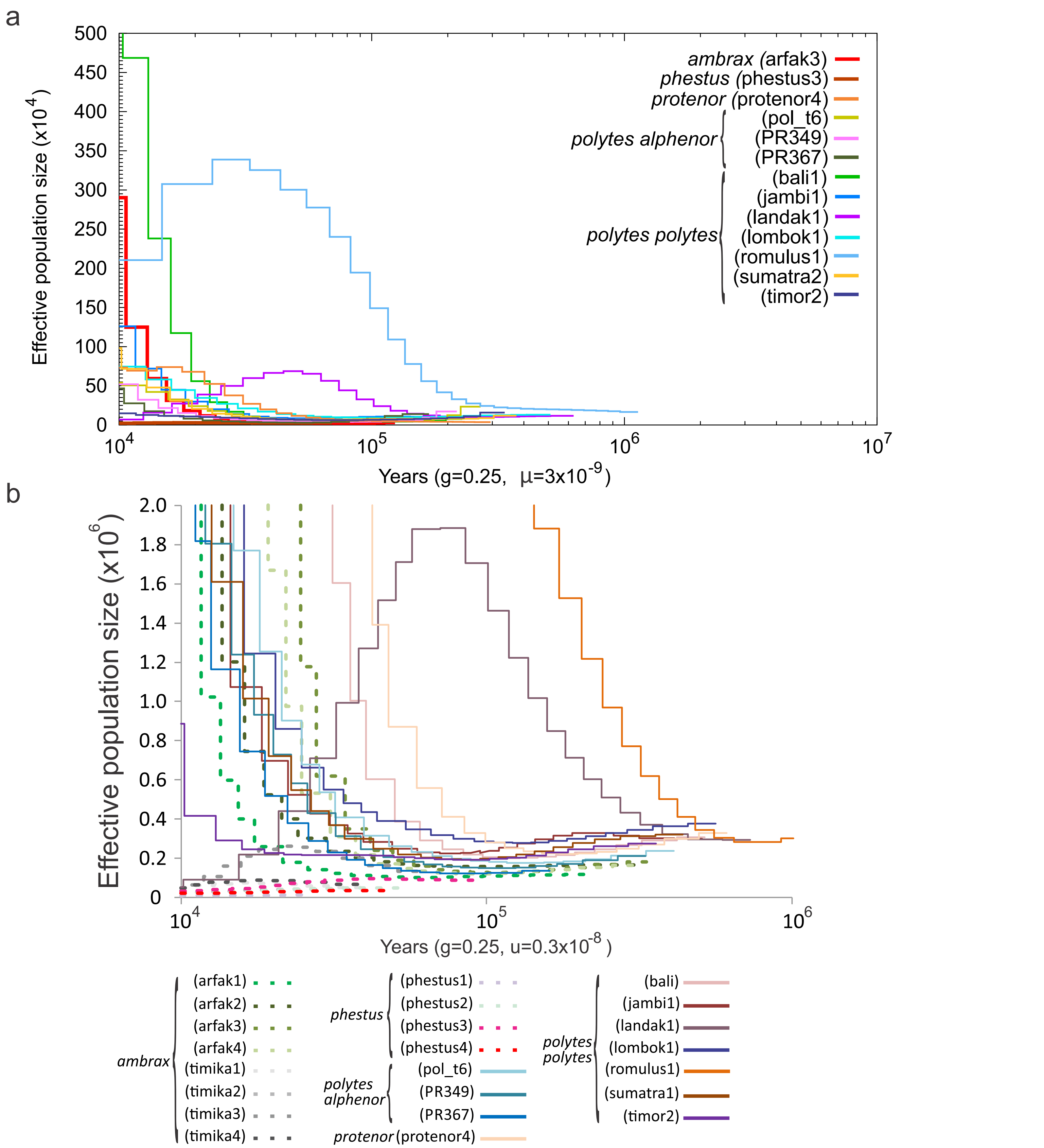

Supplementary Figure 9. Demographic history of the *polytes* species group.
(a) Historical effective population sizes were inferred from 13 individual genomes using PSMC, assuming a mutation rate of $\mu = 3.0x10^{-9}$ and an average generation time of g = 0.25 year. (b) Historical effective population sizes were inferred from 23 individual genomes using MSMC, using a mutation rate of $\mu = 3.0x10^{-9}$ and an average generation time of g = 0.25 year.

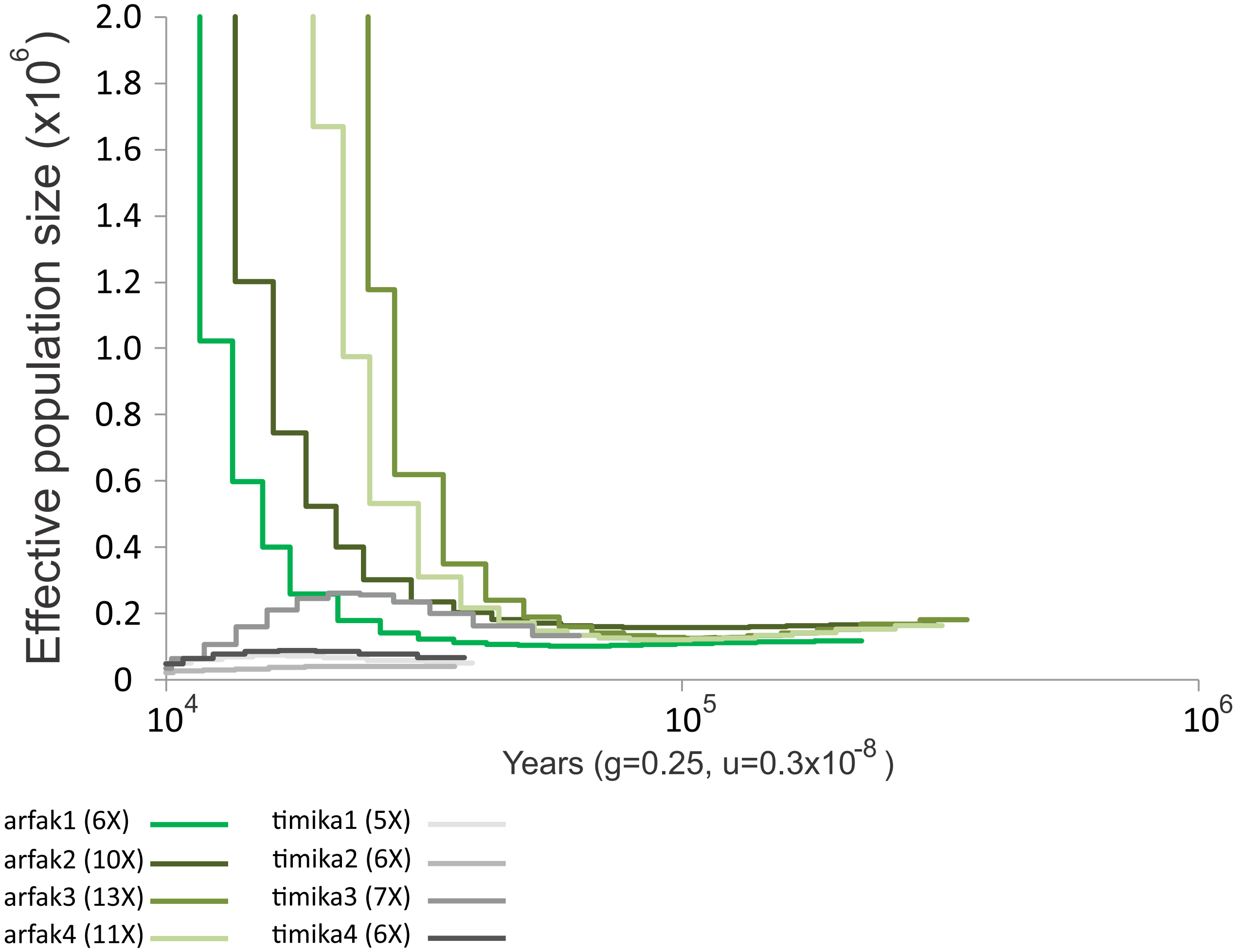

Supplementary Figure 10. Demographic history of P. *ambrax* inferred using MSMC. Historical effective population sizes were inferred from eight *P. ambrax* genomes using MSMC, using a mutation rate of $\mu = 3.0x10^{-9}$ and an average generation time of g = 0.25 year. The sequencing depth (X) is indicated for each individual.

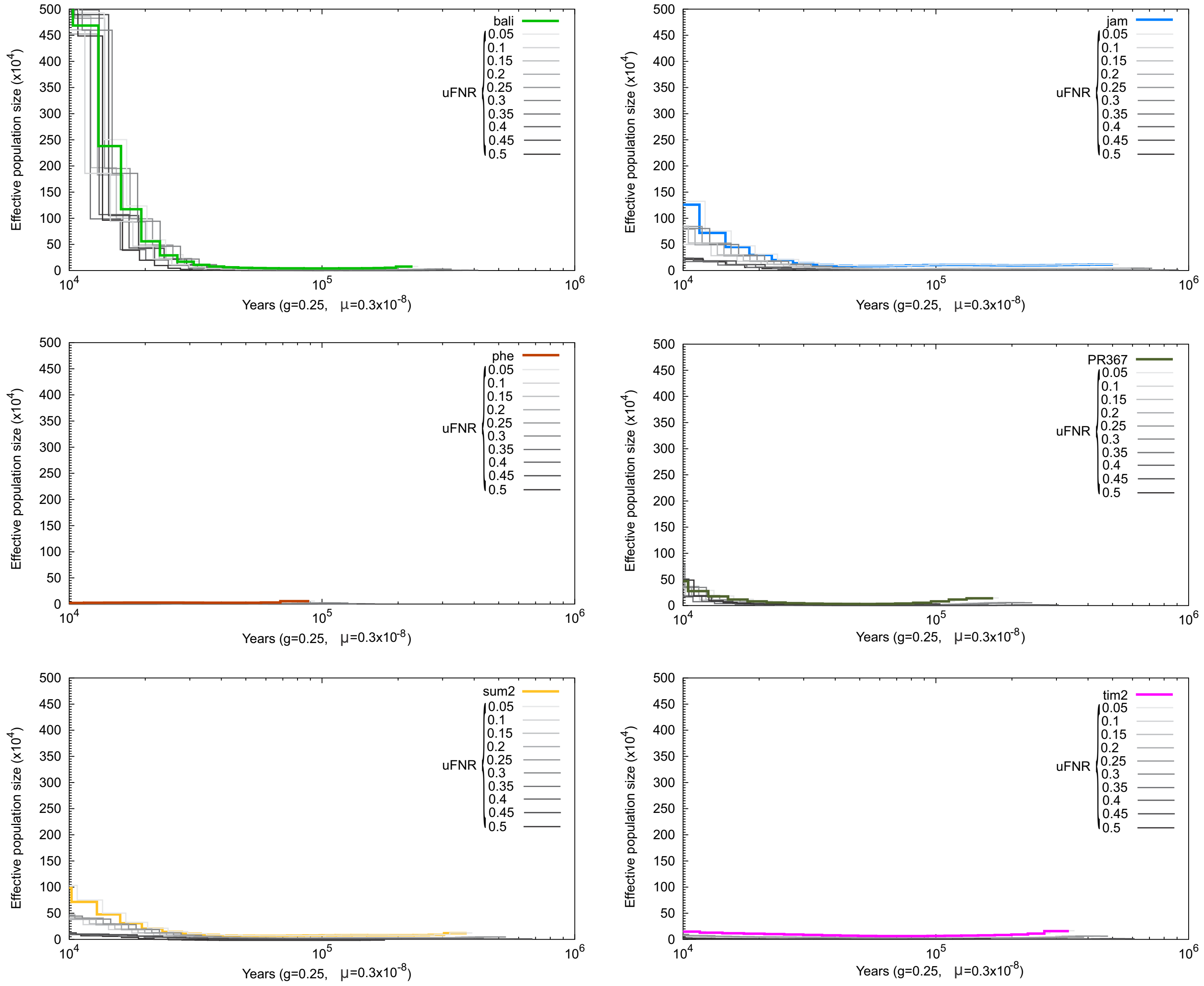

Supplementary Figure 11. Demographic history inferred using PSMC with false negative rate corrected.

Historical effective population sizes were inferred from 6 individual genomes with sequencing coverage lower than 10X using PSMC, assuming a mutation rate of $\mu = 3.0x10^{-9}$ and an average generation time of g = 0.25 year. Different values of uniform False Negative Rate (uFNR) from 0.05 to 0.5. For each individual, the original inference is plotted as a colorful line and the uFNR corrected results are plotted in grayscale.

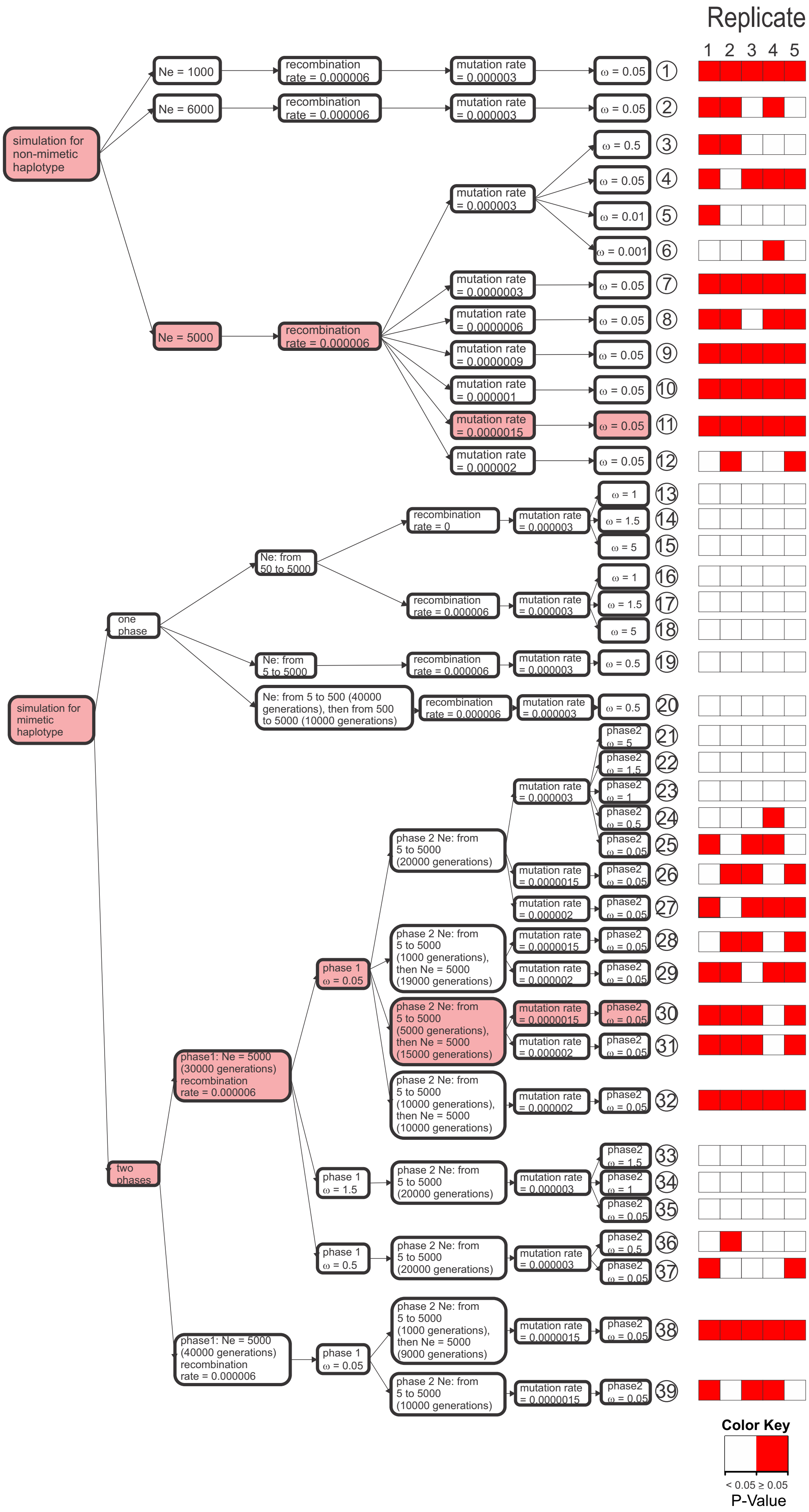

Supplementary Figure 12. Workflow for simulating the molecular evolution of the non-mimetic and mimetic *doublesex* haplotypes.
Scenario 11 and 30, which are highlighted, were selected because they yielded results most similar to our observed data. We compared empirical and simulated DNA sequence variations in *P. polytes*. Each grid represents a Fisher's exact test result from comparing a set of simulation counts with the observed variation from Fig. 4a.
